# Monkeypox Outbreak in the Democratic Republic of Congo: A Comprehensive Review of Clinical Outcomes, Public Health Implications, and Security Measures

**DOI:** 10.1002/iid3.70102

**Published:** 2024-12-16

**Authors:** Izere Salomon, Ali Emir Hamitoglu, Unkwiye Hertier, Mugabekazi Albright Belise, Uwase Sandrine, Benimana Darius, Methode Yusufu Abdoulkarim

**Affiliations:** ^1^ Department of General Medicine and Surgery, College of Medicine and Health Sciences University of Rwanda Kigali Rwanda; ^2^ Department of General Medicine, Faculty of Medicine Namık Kemal University Tekirdag Turkey

**Keywords:** clinical outcomes, Democratic Republic of the Congo, epidemiology, incidence rate, Monkeypox, public health implications, security measures

## Abstract

**Background:**

The Monkeypox virus (MPXV), a member of the *Orthopoxvirus* genus, is responsible for the zoonotic disease known as MPX. Primarily found in western and central Africa, emerging studies indicate a shift in transmission dynamics. Ongoing MPX outbreaks in the Democratic Republic of Congo (DRC) have escalated into significant public health concerns.

**Objectives:**

This review endeavors to provide a comprehensive analysis of the public health implications, clinical consequences, and preventive measures related to the current MPX outbreak in the DRC. It focuses on the epidemiology, clinical manifestations, and public health responses to this global health challenge.

**Methodology:**

The research synthesizes data regarding MPX outbreaks in the DRC, drawing from academic journals, public health reports, and case studies through a narrative review approach.

**Results:**

The recent outbreak in the DRC has identified approximately 12,569 suspected MPX cases, resulting in 581 fatalities, which corresponds to a case fatality rate (CFR) of 4.6%. These cases have been documented across 156 health sectors in 22 out of 26 provinces, representing the highest case count recorded to date. The epidemic has also encroached upon previously unaffected regions. Hospitalization rates have varied between 4% and 10%, with a significant percentage of cases attributed to sexual transmission. Analysis of demographic and geographic data revealed distinct patterns in viral spread. Clinical outcomes have varied, with an average CFR close to 10%, influenced by factors such as timely diagnosis and access to healthcare services. Rural areas have accounted for over 70% of the cases, highlighting the necessity for targeted public health interventions. Control measures have focused on community awareness campaigns and immunization programs, reaching approximately 50% of the at‐risk population; however, challenges related to resource limitations and political instability have impeded effective response strategies.

**Conclusion:**

The ongoing MPX outbreak in the DRC poses a substantial public health challenge. While progress has been made in managing the epidemic, it remains imperative to address resource deficiencies and enhance public health systems. Strengthening international collaboration, expanding healthcare access, and improving surveillance capabilities are critical to mitigating the risk of future outbreaks.

**Abbreviations**
CFR: case fatality ratio
COVID‐19: coronavirus disease 2019
DRC: Democratic Republic of the Congo
MPX: monkeypox
MPXV: monkeypox Virus
PCR: polymerase chain reaction
RT‒PCR: real‐time polymerase chain reaction
STIs: sexually transmitted Infections
WHO: World Health Organization

## 1 Introduction

Monkeypox, formerly known as Mpox, is a zoonotic illness caused by a viral species that is part of the *Orthopoxvirus* genus, *Chordopoxvirinae* subfamily, *Poxviridae* family, and *Orthopoxvirus* genus. The Mpox virus, an Orthopoxvirus belonging to the same genus as the variola virus that causes smallpox, vaccinia, and cowpox, is the cause of this zoonotic viral disease. Infection caused by the Mpox virus, which causes a vesicular rash resembling smallpox, fever, headache, and lymphadenopathy. Nonetheless, case mortality rates and person–person transmission beyond intimate contacts vary from 1% to 10%, contingent upon the virus strain and host immunological status, and monkeypox infection is significantly less common than smallpox infection [1, 2]. The identification of MPXV dates back to 1958, when a nonfatal rash disease struck captive cynomolgus monkeys in Copenhagen, Denmark. The Mpox virus was first detected in 1970, 9 months after the Democratic Republic of the Congo (DRC) declared smallpox extinct [3, 4]. Since then, monkeypox outbreaks have occurred in laboratories and zoos, and human cases have been reported in Central and Western Africa [5, 6, 7, 8]. Human‐to‐human transmission has been observed in Central Africa [9], and there have also been outbreaks in nonendemic countries linked to imported animals, such as the 2003 outbreak in the United States [10]. These outbreaks highlight the global importance of this emerging zoonosis [11].

**Figure 1:**
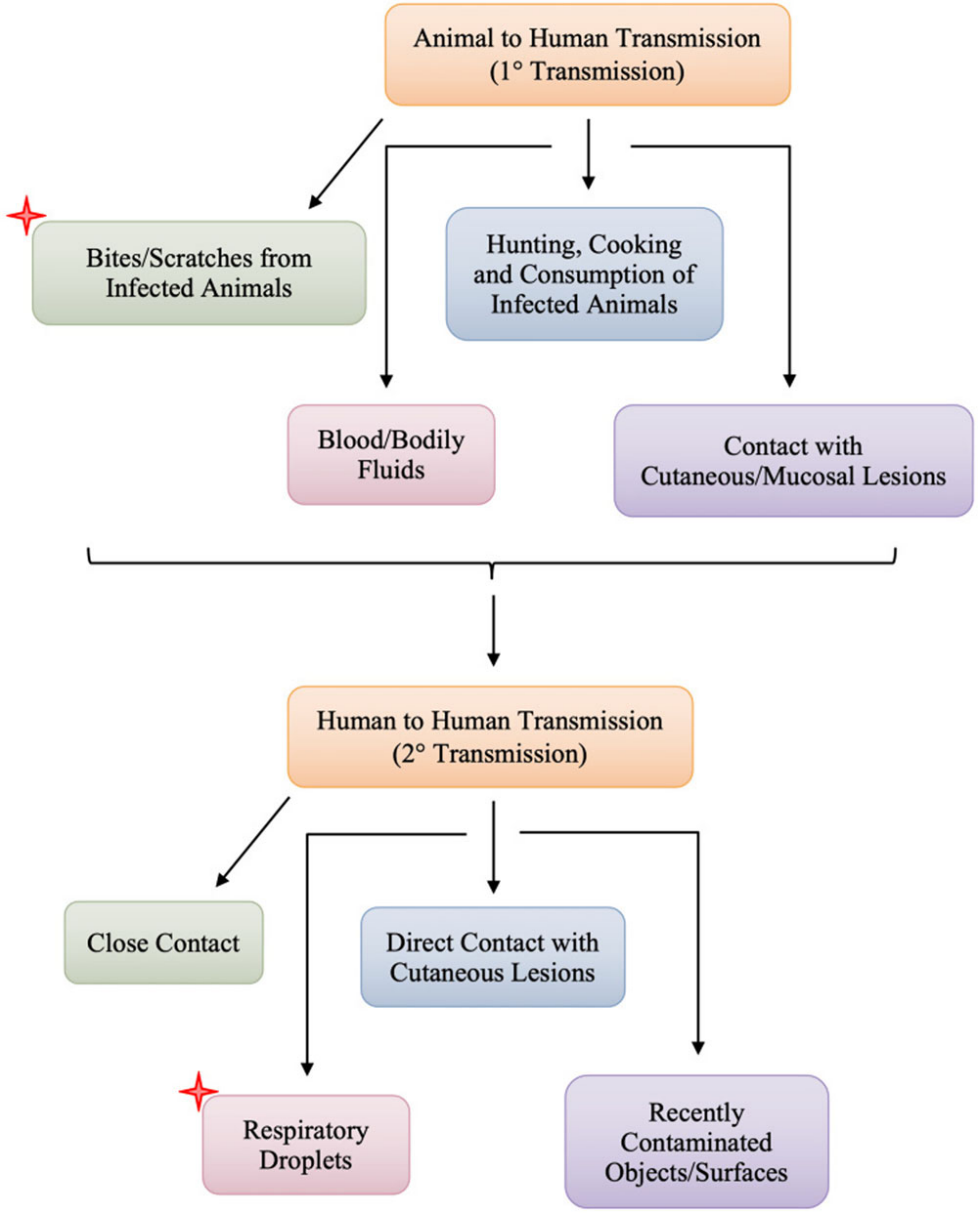
An illustration of the transfer of the Mpox virus from humans to the environment [12]. The red asterisks emphasize critical transmission pathways.

MPXV can be transmitted through direct contact with infected animals or humans, as shown in Figure 1 [13]. Animal‐to‐human transmission occurs through bodily fluids, such as saliva or respiratory excretions, and through exposure to infected animal feces [12]. Due to limited resources, visiting forests where infected animals are common or sleeping outdoors, increases their risk of exposure [8]. Human‐to‐human transmission is less common and involves prolonged face‐to‐face contact or contact with infected lesions [14]. Recent studies highlight the prevalence of concurrent sexually transmitted infections (STIs) among mpox patients indicating that Mpox is also transmitted through sexual contact, with a high frequency of anogenital lesions suggesting direct inoculation during sexual activities. In a Spanish study, 30 out of 157 mpox patients tested positive for various STIs, including herpes simplex virus, Neisseria gonorrhoeae, and Chlamydia trachomatis [15]. Similarly, a US study found that 15% of patients tested for mpox were diagnosed with concurrent STIs [16]. Additionally, The most extensive study across 16 countries reported a 29% rate of concomitant STIs among mpox patients, with syphilis, gonorrhea, and chlamydia being the most common [17]. These findings underscore the importance of comprehensive STI screening for mpox patients, particularly those with high‐risk sexual behaviors [18]. Emphasizing the need for comprehensive STI screening in mpox management [19]. Both human‐to‐human and animal‐to‐human virus transmission determine the etiology and pathophysiology of the illness.

**Figure 2:**
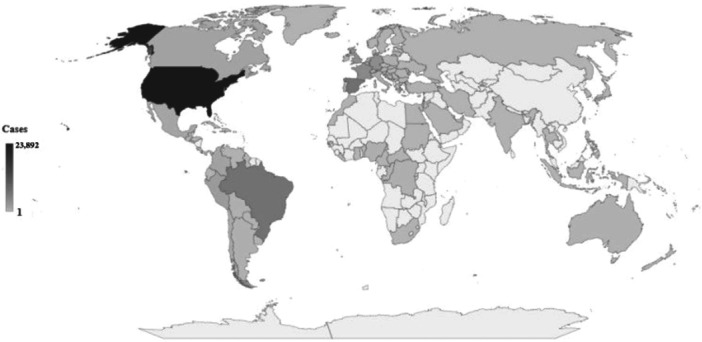
Global map of the human monkeypox outbreak as of September 19, 2022, showing the total number of confirmed cases by nation [20].

Since May 2022, there have been more than 62,000 reported cases of human monkeypox globally, with 24,017 cases identified across 44 European countries. The countries in Europe with the highest incidence of cases include Spain, France, Germany, and the United Kingdom (Figure 2) [20]. The outbreak has revealed sustained person‐to‐person transmission and exhibits distinct clinical characteristics compared to prior studies. Hospitalization rates are reported to range from 4% to 10%, and the condition is categorized as mild to moderate. To date, there have been reports of 20 fatalities, originating from both nonendemic and African countries. Notably, the documented prevalence of numerous sexual partners and incidents of chemsex indicate that sexual transmission among men who have sex with men is a significant factor contributing to the outbreak. The vaccination status of the affected individuals varies, with several healthcare professionals contracting the infection in the course of their duties. Furthermore, individuals aged 40 to 50 years face a heightened risk of infection, as smallpox vaccination campaigns have ceased. In addition to that, More investigations are needed to determine whether the virus can be transferred via genital secretions during sexual activity [8, 19, 21, 22, 23, 24, 25, 26, 27, 28, 29, 30]. The purpose of this review is to present a thorough examination of the outbreaks of mpox in the DRC, emphasizing the clinical results, public health responses, security measures, and suggestions to reduce the spread of the illness and prevent such outbreaks in the future.

## 2 Recent Mpox Outbreaks in the DRC

A recent mpox outbreak began on January 1 in the DRC. A total of 12,569 suspected cases and 581 deaths were reported in 156 health zones in 22 out of 26 provinces [19]. With additional cases appearing in places such as Kinshasa, Lualaba, and South Kivu that had not previously reported mpox, this outbreak represents the greatest number of cases ever recorded. It has been noted that human‐to‐human transmission in nonaffected provinces has been fueled by cases with travel histories to endemic regions [19].

In Kenge, Kwango Province, the first cases of sexually transmitted mpox were also reported during this outbreak. The first case that is known to exist involved a Belgian‐born man who traveled to Kinshasa and soon after she began to exhibit symptoms. Clade I MPXV was the virus that was identified by epidemiological studies, and it was most likely spread in Belgium. Several sexual and nonsexual contacts were positive for mpox after additional investigations [19].

Furthermore, outbreaks of mpox were observed in South Kivu and Kinshasa, where no cases had been recorded before. While the initial case in South Kivu involved a young trader who traveled from a province where mpox was endemic, Kinshasa experienced local transmission from people exposed in other areas. The province is now dealing with several issues that could contribute to the further spread [19].

In the DRC, research on animals has been carried out to learn more about the ecology and possible hosts or reservoirs of the mpox virus. To determine the virus reservoir and accidental hosts, national and international research partners are working together on these ongoing studies [19].

## 3 Epidemiology

Central Africa's largest country, the DRC, has a population of approximately 102.3 million [31]. The topography of the nation is varied and includes mountains, grasslands, savannas, tropical rainforests, and plateaus. There are 26 provinces in the country, and each has its own administrative and healthcare systems. Poverty, violence, relocation, inadequate health systems, and frequent outbreaks of infectious diseases such as cholera, measles, and Ebola are just a few of the numerous difficulties it faces [31].

Mpox, previously referred to as monkeypox, is a disease that is transmitted from animals to humans and is caused by the mpox virus, which belongs to the *Orthopoxvirus* genus [3, 19, 29]. Clade I, formerly known as the Congo Basin clade, and clade II, formerly known as the West African clade, which were formerly found in Central and West Africa, respectively, make up the MPXV genetic group. Clade IIa and Clade IIb are the two subclades that make up clade II. The global mpox outbreak that began in 2022 and has impacted numerous non‐African countries is the result of clade IIb [19, 31, 32, 33, 34].

Since the disease is endemic and has been documented since 1970, the DRC has been among the countries most impacted by mpox worldwide. Fever, headache, sore muscles, enlarged lymph nodes, and a vesicular‐pustular rash that may leave scars and scabs are the disease hallmarks [23, 30]. Through direct contact with bodily fluids, lesions, or contaminated objects, the disease can spread from humans to animals or from animals to humans. There have also been reports of sexual transmission, particularly between men and guys who have intercourse [19, 21, 22].

**Figure 3:**
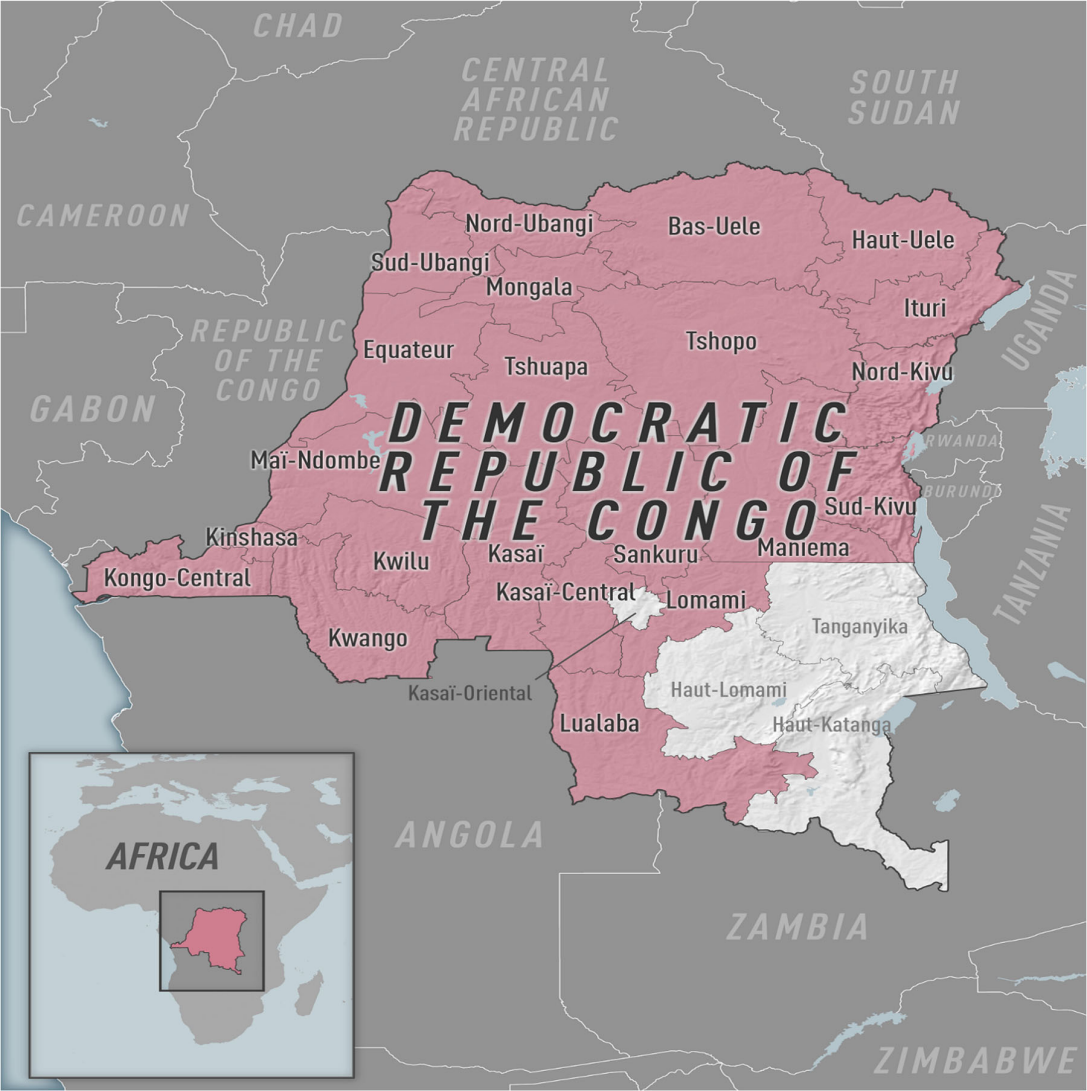
The DRC's map area. Provinces with confirmed and probable cases of mpox are shaded in pink [35].

**Figure 4:**
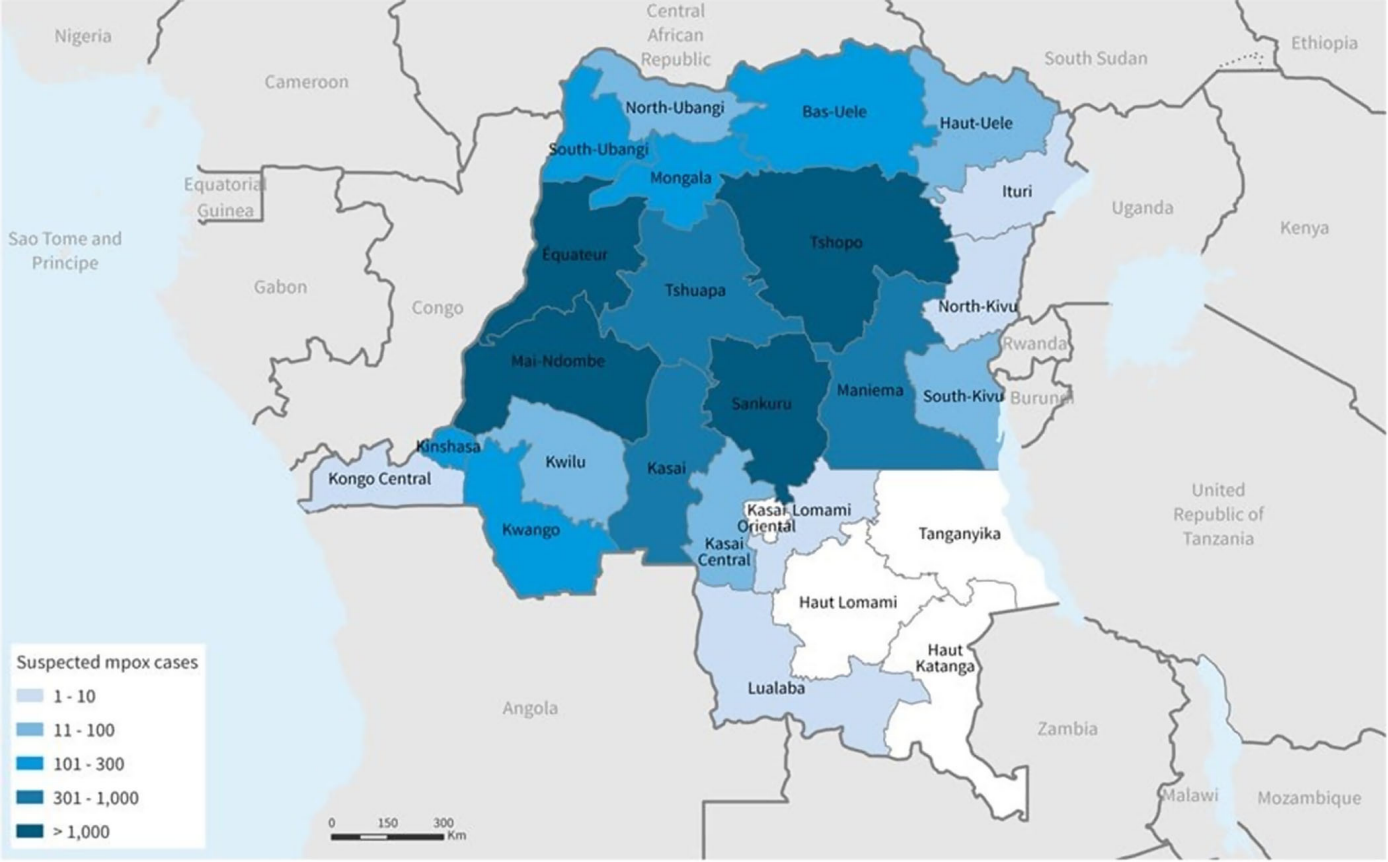
Locations of suspected cases of mpox by DRC province, January 1–November 4, 2023 (Epi weeks 1–44) [19].

**Figure 5:**
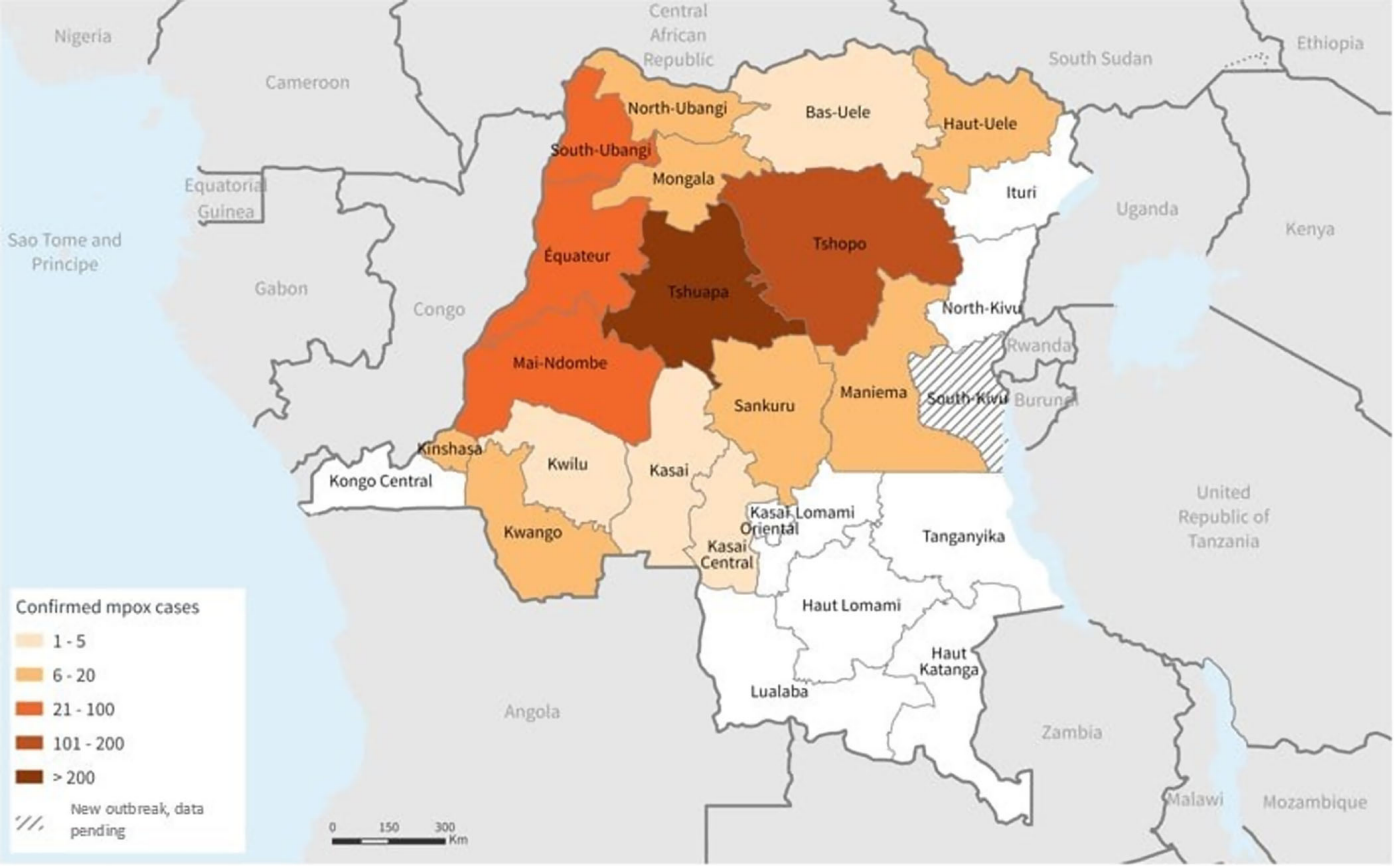
Location of suspected mpox cases in the Democratic Republic of the Congo Province, January 1, 2023–October 7, 2023 (Epi weeks 1–40) [19].

Eleven provinces, mostly in the country's north and central regions, are endemic for the disease. In these areas, the virus spreads among wildlife reservoirs, including rodents and primates. Nonetheless, the number of provinces reporting mpox has increased to 22 in recent years (Figure 3), including areas that had not previously reported mpox, such as South Kivu, Lualaba, and Kinshasa (Figures 4 and 5) [19, 35].

With more than 12,000 probable cases and more than 300 confirmed deaths since January 2020, the current mpox outbreak in the DRC is the largest and longest‐lasting outbreak in the history of the Country [19, 36]. MPXV clade I, which is endemic to the Country and has not been linked to the global outbreak of clade IIb, is the cause of the outbreak. However, incidences of clade I virus transmission through sexual contact as well as cases involving sex workers have been reported for the first time, indicating a possible alteration in the virus's transmission dynamics [19].

According to Table 1 data, the outbreak worsened from 2018 to its peak point in terms of cases, fatalities, and case‐fatality ratios (CFR) in 2023. There is a greater risk of infection and transmission in the population, as evidenced by the increase in both attack rates and reproductive numbers.

**Table 1:** Primary epidemiological markers of the monkeypox outbreak in the DRC from 2018 to 2023 [19] according to WHO statistics.

Year	Suspected cases	Deaths	CFR	Attack rate	Reproductive number
2018	2850	60	2.1%	3.2/100,000	0.7
2019	3794	73	1.9%	4.2/10,000	0.8
2020	6216	222	3.6%	6.9/100,000	1.2
2021	5784	81	1.4%	6.4/100,000	0.9
2022	8432	156	1.9%	9.4/100,000	1.1
2023	12,569	581	4.6%	14.0/100,000	1.4

## 4 Clinical Presentation and Diagnosis of Mpox

The symptoms of the monkeypox virus are difficult to detect since they resemble those of other common illnesses, leading to a long list of differential diagnoses [25, 35]. Pregnant women, immunosuppressed individuals, and children under the age of 15 are among the groups most adversely impacted by the disease; however, some individuals may only show moderate symptoms that seldom necessitate medical attention [19]. The WHO reports that skin rashes are the most typical signs of the human mpox virus. These rashes can also include fever, headaches, muscle aches, back discomfort, low energy, and swollen lymph nodes [19]. There is a chance of transmission before mpox is identified because the incubation period is thought to be between 5 and 21 days, and the symptoms and signs persist between 2 and 5 weeks. Numerous studies have delineated the phases of MPX progression, separating it into two primary stages. The first phase lasts 1 to 5 days and is characterized by lymphadenopathy, fever, headache, back discomfort, and low energy. The second stage, which is marked by a rash, occurs approximately 1 to 3 days after the fever has gone down [33, 37, 38].

Although MPX belongs to the large family of *poxviruses*, its distinctive symptom of lymphadenopathy sets it apart from the others and facilitates diagnosis [33]. The WHO advises using a PCR test of viral swabs as a confirmatory test for monkeypox diagnosis; however, for samples obtained from vesicles, it is crucial to ensure that all protocols are followed to optimize the test's efficacy, including appropriate sample collection and laboratory transportation [19, 39, 40]. However, this strategy does not eliminate the need for additional techniques, such as viral cell separation by culture or immunological techniques.

Each diagnostic technique has pros and cons and finding affordable tools that can meet the demands of mass screenings, speed, sensitivity, and specificity, among other requirements, can be challenging. Depending on the settings, popular suggestions include pairing techniques to compensate for the absence of any of the capabilities.

According to recent studies, there is a greater risk of the virus spreading from person to person. Perianal vesicular symptoms are more common, indicating a sexual channel of transmission. This is particularly true for men who have sex with other men, with a high frequency of anogenital lesions suggesting direct inoculation during sexual activities. Concurrent STIs are common among mpox patients, with various pathogens detected, including herpes simplex virus, Neisseria gonorrhoeae, and Chlamydia trachomatis [17, 18, 21, 22, 33]. More evidence‐based research in this area is now more important than ever, and to prevent misdiagnosis errors, new diagnostic methods with far higher specificity and sensitivity than those currently in use must be developed.

Accurate diagnosis, capacity building in affected areas, international collaboration, and community education should be given top priority when allocating funds for research. To contain monkeypox outbreaks and improve global readiness for emerging infectious hazards, a comprehensive approach integrating robust surveillance, enhanced international cooperation, and efficient diagnostics is needed. Resources must be directed toward the development of accurate and accessible diagnostic technology. A comprehensive understanding of the clinical presentation and diagnostic challenges associated with monkeypox is crucial for a timely and effective epidemic response. To overcome current obstacles and strengthen global preparedness against newly emerging infectious risks, advancements in diagnostic methods and ongoing research are needed.

## 5 Public Health Responses

A nationally funded strategy for Mpox preparation and response was created by the DRC Ministry of Public Health, Hygiene, and Prevention. It included a variety of tasks that needed to be carried out [19, 35]. monitoring and detection strategies include stepping up mpox surveillance nationwide, especially in Kinshasa; Kenge, and Kamituga; providing reference hospitals with sample collection and transport kits and logistical support for gathering, transporting, and analyzing samples from suspected cases from affected areas; enhancing the capacity of healthcare providers and professionals in mpox surveillance; keeping track of contacts of clinical and laboratory‐confirmed cases for 21 days; genetically sequencing mpox samples to better understand circulating viral strains; and educating groups potentially at risk of danger.

Case management, infection prevention, and control, which include equipping medical personnel in the most affected areas with personal protective equipment and isolating confirmed cases in hospitals or at home to stop the spread of the infection; and training and capacity building, which includes educating laboratory personnel in Kinshasa and other affected areas or provinces on how to collect, preserve, and properly deliver mpox samples [19, 39].

However, the most recent WHO situation report indicates that there are still some gaps and challenges in the public health response to the monkeypox outbreak in the DRC. These include the persistence of the outbreak in some provinces, particularly in the Mai‐Ndombe Region, where the case fatality ratio is above 10% and transmission is high; the emergence of sexual transmission of monkeypox, which presents a new risk factor and complicates contact tracing and prevention [19, 41]; the restricted accessibility and availability of the vaccine, which hinders the campaign's coverage and equity, particularly in rural and difficult‐to‐reach areas; the underdeveloped health system, which is overburdened with numerous public health emergencies (such as COVID‐19, measles, and Ebola) and lacks sufficient infrastructure, personnel, supplies, and equipment; the low level of community engagement and trust, which influences the adoption and adherence of public health interventions; and the requirement for more specialized and culturally aware communication strategies [41].

## 6 Challenges and Lessons for Future Outbreak Preparedness

In addition to presenting several difficulties for the public health response, the monkeypox outbreak in the DRC has taught important lessons for future disease prevention and management. Three primary issues were recognized and examined, along with the pertinent takeaways from the monkeypox pandemic.

## 7 Limited Resources

With a poor health system and low income, the DRC is confronted with several public health emergencies, including COVID‐19, measles, and Ebola, which put further strain on its already constrained resources [42]. A lack of resources, staff, equipment, and supplies has complicated the response to the monkeypox outbreak, particularly in isolated and rural areas where the illness is endemic [43]. The quality and promptness of laboratory, immunization, surveillance, and infection prevention and control (IPC) operations, as well as the delivery of sufficient patient and community care, have all been impacted by the lack of resources [19, 44, 45]. Consequently, in addition to prioritizing and coordinating the response to monkeypox with other existing public health activities, there is a need to enhance the health system and mobilize additional resources. This could entail increasing the amount of money that is sought after from partners and donors, providing additional material and human resources to the impacted areas, fostering better coordination and collaboration between many stakeholders, and strengthening the ability and resilience of healthcare providers and facilities.

## 8 Logistical Difficulties

The Democratic Republic of the Congo is a vast and heterogeneous nation with intricate topography and inadequate facilities, creating logistical challenges for the distribution and execution of public health initiatives. Transporting and distributing the vaccine, laboratory reagents, and personal protective equipment and getting to and reaching the afflicted individuals, particularly in remote and unsafe locations, have been difficult in response to the monkeypox outbreak [19, 24, 45]. To guarantee the safety and accessibility of response teams and supplies, it is necessary to enhance supply chain management and logistics as well as work in tandem and coordinate with local authorities and partners, including the military, the police, and humanitarian organizations. This could entail putting in place more dependable and efficient methods for communication and transportation, ensuring that supplies are stored and maintained appropriately, creating backup plans in case of emergencies, and cultivating a relationship of trust and collaboration with the local authorities and community.

## 9 Community Engagement

The Democratic Republic of the Congo is a diverse and dynamic culture with a wide range of political, religious, and cultural practices and beliefs. These factors impact how communities perceive and respond to disease. Engaging and organizing communities to take part in and adhere to public health actions, such as reporting cases, accepting immunization, and minimizing contact with animals, has been difficult in the wake of the MPX outbreak [46, 47]. In addition, historical, political, and societal elements such as the effects of colonization, ongoing hostilities, and false information and rumors have caused opposition and mistrust in some areas. In addition to involving and empowering community‐based structures such as health committees, community health workers, and community leaders who can facilitate dialog and feedback between health authorities and communities and encourage the adoption of positive health behaviors, more specialized and culturally sensitive communication strategies need to be developed and put into practice. This could entail developing and delivering clear and consistent messages through appropriate channels and languages, addressing myths and misconceptions about the disease and the response, conducting formative research to understand the local context and needs, and acknowledging and rewarding the contributions and accomplishments of the communities.

## 10 Recommendations and Future Directions

When MPX outbreaks occur in nonendemic areas, efficient containment and treatment measures are needed [47]. There are no specific treatments for MPX, and little is known about its pathophysiology and epidemiology. For prevention, two smallpox vaccines, ACAM2000 and JYNNEOSTM, can be used as pre‐exposure prophylaxis, with JYNNEOSTM also approved for post‐exposure use. Ongoing research is focused on developing more effective and safe treatments for mpox [33, 48]. PCR technology is utilized to diagnose MPX, and in more severe cases, antiviral medications may be prescribed [19, 49]. Its control measures include immunizing close contacts, stopping the spread of the illness, and promptly identifying and isolating cases. Further information is required about the effectiveness and safety of these therapies in treating MPX infections. To prevent and control MPX outbreaks, international collaboration, immunization programs, and surveillance are essential. To lessen the impact of future outbreaks, preparedness measures, including sound monitoring and detecting systems, are needed. To investigate variations in clinical features and their causes, more research is needed.

The DRC's current 2023 MPX outbreak necessitates a number of crucial measures [19, 50, 51]. First, new epidemic investigations and response initiatives should be put into place, along with increased national surveillance. This entails being alert, quickly identifying and reporting symptoms, and isolating patients. Second, to diagnose and isolate suspected or confirmed cases as soon as possible, healthcare personnel should receive diagnostic tool training and equipment. Third, there should be more cross‐border cooperation and data exchange on infectious diseases, including human MPX. Fourth, a “one health” strategy that prioritizes handwashing, infection prevention, and cleanliness in hospitals should be implemented. WHO and other international health organizations should assist the healthcare sector in efficiently handling the outbreak.

## 11 Conclusion

The growing number of infections and the burden on the health system make the 2023 MPX outbreak in the DRC a concerning situation. In comparison to earlier outbreaks, a significant number of new outbreaks have been revealed. This highlights the need for surveillance, immunization, and international collaboration, as well as the need for additional research and advancements in the management and prevention of MPX. Important tasks include creating and assessing novel MPX diagnostics, therapies, and vaccinations; determining the effectiveness and financial implications of public health initiatives; and putting preventative measures in place to stop such outbreaks in the future. The outbreak has provided the public health community with an opportunity to learn since it has highlighted the shortcomings and shortcomings of the response as well as the gaps and requirements for the future.

## Author Contributions

**Izere Salomon:** conceptualization, methodology, project administration, supervision, validation, writing original draft, reviewing draft, and correspondence. **Ali Emir Hamitoglu:** writing–review and editing. **Unkwiye Hertier:** writing–review and editing. **Mugabekazi Albright Belise:** writing–review and editing. **Uwase Sandrine:** writing–review and editing. **Benimana Darius:** writing–review and editing. **Methode Yusufu Abdoulkarim:** writing–review and editing.

## Conflicts of Interest

The authors declare no conflicts of interest.

## Data Availability

The authors have nothing to report.
